# New insights into multistability and complex resonances driven by subthreshold periodic signals in a neuronal model

**DOI:** 10.1007/s11571-025-10358-3

**Published:** 2025-10-24

**Authors:** Agustín Farrera Megchun, Pablo Padilla-Longoria, Jesús Espinal-Enríquez, Gerardo J. Escalera Santos, Roberto Bernal-Jaquez

**Affiliations:** 1 Posgrado en Ciencias Naturales e Ingeniería, Universidad Autónoma Metropolitana-Cuajimalpa, Av. Vasco de Quiroga 4871, Santa Fe Cuajimalpa, 05348 Mexico City, Mexico; 2 Instituto de Investigaciones en Matemáticas Aplicadas y en Sistemas, Departamento de Matemáticas y Mecánica, Universidad Nacional Autónoma de México, 04510 Mexico City, Mexico; 3 Computational Genomics Division, National Institute of Genomic Medicine, 14610 Mexico City, Mexico; 4 Facultad de Ciencias en Fisica y Matematicas, Universidad Autonoma de Chiapas, 29050 Tuxtla Gutiérrez, Chiapas Mexico; 5 Departamento de Matematicas Aplicadas y Sistemas, Universidad Autonoma Metropolitana-Cuajimalpa, Av. Vasco de Quiroga 4871, Santa Fe Cuajimalpa, 05348 Mexico City, Mexico

**Keywords:** Huber-Braun neuron, Signal detection, Multistability, Nonlinear resonance, Basins of attraction, Energy consumption

## Abstract

Understanding how neurons respond to weak external signals is crucial for accurate signal transmission and processing in both individual nerve cells and interconnected neuronal networks. One mechanism for the detection of these responses is through resonances. In this paper, we numerically investigate the firing patterns induced in a silent Huber-Braun neuron by a sinusoidal external force. We observe complex resonance patterns, including a sequence of frequency-locking exhibited in a Devil’s Staircase structure. Furthermore, we also explore the emergence of multistability induced by the nonlinear resonance. This multistability manifests as the coexistence of three attractors, such as periodic spiking, chaotic spiking, and subthreshold oscillations. The dynamical behaviors are comprehensively analyzed using time series, bifurcation diagrams, phase portraits, and the basin of attraction. In addition, we compute the maximum Lyapunov exponent to verify chaotic regimes, and estimate the fractal dimension of basin boundaries using the uncertainty exponent. We also analyze the energy consumption of resonance-induced firing patterns and coexisting attractors. The results presented in this paper have important implications for understanding the detection of subthreshold signals and the encoding of stimulus information within a neuron’s firing patterns.

## Introduction

Neurons, the fundamental units of the nervous system, exhibit a remarkable ability to selectively process information from their environment and react with complex responses to external stimuli. A key mechanism underlying this selectivity is resonance. Resonance describes a phenomenon where an external force, applied at a specific frequency, induces a system to oscillate with a significantly bigger amplitude. In the nervous system, resonance is a measurable property that describes the ability of neurons to respond selectively to inputs at preferred frequencies and may selectively translate to spiking patterns (Hutcheon and Yarom 2000). For instance, in the hippocampus, different types of neurons have distinct frequency preferences; when sinusoidal currents are applied; pyramidal neurons show a firing preference at theta frequencies (2–7 Hz) (Pike et al. 2000).

Mathematical models of neurons provide a powerful tool for investigating the underlying mechanisms of these phenomena from the perspective of nonlinear dynamics. The concept of resonance has been extensively studied in nonlinear systems, spanning from the nonlinear resonance observed in deterministic systems to stochastic resonance arising from the presence of noise (Liu et al. 2020; Parmananda et al. 2005, 2002; Santos et al. 2004; Palabas et al. 2023; Rajasekar and Sanjuan 2016). When injected a periodic current, neurons exhibit complex firing activities, for example, mode locking is observed in periodically stimulated neurons such as giant squid axons (Aihara et al. 1985; Matsumoto et al. 1987; Takahashi et al. 1990). In response to an external forcing, nonlinear systems exhibit complex behaviors characterized by *p* : *q* frequency-locking (i.e., an oscillation of action potential *p* generated by *q* cycle stimulation). This behavior creates a sequence of steps known as the Devil’s Staircase. The *p* : *q* frequency-locking, also known as *p* : *q* synchronization, gives rise to synchronous regimes of order *p* : *q*. These regions, now commonly called Arnold tongues, delineate the parameter space where specific synchronization patterns dominate. When multiple *p* : *q* sequences are observed, this phenomenon is referred to as higher-order synchronization and it has been observed in a variety of nonlinear systems (Pikovsky et al. 2001), including van der Pol oscillators (Parlitz and Lauterborn 1987), pendulums (d’Humieres et al. 1982), and lasers (Simonet et al. 1994). Additionally, it is prevalent in real biological systems such as embryonic heart cell aggregates (Guevara et al. 1981), sinoatrial node cells (Anumonwo et al. 1991), and the giant axons of squid (Takahashi et al. 1990).

Another important aspect of periodic perturbation in neurons is the ability to induce multiple coexisting attractors in both coupled and individual neurons (Bosco et al. 2024; Manchein and Rech 2024; Njitacke et al. 2021). Multistability is a complex dynamical phenomenon in which a nonlinear system with fixed parameters can display different stable behaviors depending on its initial conditions. Specifically, it refers to the coexistence of three or more dynamical behaviors. In recent years, the study of multistability has gained considerable attention, not only in neuron models but also in various physical systems where the effects of external stimuli are significant, such as metasurfaces, torsional vibrations, and nonlinear damping gyroscopes (Leutcho et al. 2023; Meli et al. 2021; Miwadinou et al. 2020). In neurons, state transitions due to multistability may underlie mechanisms for information storage and processing (Canavier et al. 1993), and this phenomenon has been observed in neuron R15 in the abdominal ganglion for *Aplysia* (Canavier et al. 1994). Recent memristive neural networks explicitly link multistability to dynamic memory/information storage and demonstrate coexisting attractors and rich multiscroll dynamics, with practical roles in decision circuits and signal processing (Gonzalez et al. 2025; Wang et al. 2025a; Zhang et al. 2024a; Wang et al. 2025b). The study of multistability has received considerable attention in various neuron models (Ngouonkadi et al. 2016; Stankevich and Mosekilde 2017; Peng et al. 2023; Fossi et al. 2023; Li et al. 2024a), for example, in an active memristive neuron model using the 2D Hindmarsh and Rose (HR) neuron model, multistable phenomena of four coexisting firing patterns are generated (Lin et al. 2020a). Moreover, a periodic stimulus injected in the HR neuron model induced coexisting behaviors of asymmetric bursters (Bao et al. 2018). Recent studies indicate that external stimulation can induce the coexistence of multiple attractors in low-dimensional, non-spiking recurrent neural networks (Zhang et al. 2024b; Hua et al. 2022; Doubla et al. 2022; Lin et al. 2020b; Vignesh et al. 2024). Additionally, nonlinear resonances and multistability have been observed in simple neural circuits (Alonso 2017).

Recent studies in neuron models have provided new insights into how periodic external forcing shapes spiking responses (Boaretto et al. 2025), giving rise to complex mode-locking patterns (higher-order synchronization) (Abhay and Dar 2025), and the coexistence of distinct firing regimes (Li and Li 2025). In particular, efforts have also been made to investigate neuron behavior from the physical perspective, including circuit implementations (Ma 2025). For example, a second-order RC-oscillator-based piecewise linear neuron circuit exhibits the coexistence of multiple attractors (Chen et al. 2025), and nonlinear resonance has been reported in neurons driven by memristive currents (Wang et al. 2025c). These achievements underscore that periodic perturbations, both in mathematical models and physical circuits, can fundamentally reorganize neuronal dynamics, motivating our focus on the Huber–Braun model under subthreshold forcing.

In this work, we study a neuron model when a sinusoidal external current is superimposed on the system, where the current is restricted to only oscillating in the fixed point region. When the external amplitude is zero, the neuron becomes silent. Previous studies have shown that a subthreshold sinusoidal current near a Hopf bifurcation can induce spiking dynamics via nonlinear resonance (Parmananda et al. 2001, 2002). This phenomenon arises from the interplay between the frequency of the sinusoidal perturbation and the characteristic frequency of the damped oscillations around the stable fixed point. Such currents can lead to various mode-locked states in the Hodgkin-Huxley model, including bistability regions (Lee and Kim 2006). Moreover, they can evoke spiking activity in the Morris–Lecar (Xie et al. 2005) and Hindmarsh-Rose (Wang et al. 1998) models.

While multistability has been investigated in various neuron models, its emergence under subthreshold periodic stimulation remains a key area requiring further investigation. In this work, we address this gap using a biologically realistic, conductance-based neuron model known as the Huber–Braun (HB) model. Although the periodically forced HB model has been explored in previous studies (Wang et al. 2015; He et al. 2023), including a recent one reporting the emergence of a periodic structure–termed a “fishbone”–within chaotic dynamics (Boaretto et al. 2025), our work focuses on an aspect that, to the best of our knowledge, has not been analyzed in detail: the emergence of multistability and complex resonances under the action of subthreshold periodic stimuli. This is a nontrivial and biologically relevant extension of the classical model that may help explain the sensitivity and complex switching behavior of thermoreceptive neurons in response to weak external inputs.

The HB model is a modified version of the Hodgkin–Huxley model (Braun et al. 1998), which has been developed to simulate the impulse activity of peripheral cold receptors in response to temperature changes. This model exhibits an enormous variety of different impulse patterns observed in electroreceptors from dogfish, catfish, facial cold receptors, and hypothalamic neurons of the rats (Braun et al. 1994, 1997, 1999). In recent years this model has been used in a variety of contexts, for example, noise effects (Finke et al. 2010a, 2008), tonic-bursting transitions (Shaffer et al. 2016, 2017), phase synchronization (Prado et al. 2014; Budzinski et al. 2019), effects of neuron position (Farrera-Megchun et al. 2024, 2025a), spiral waves in multilayer network (Rajagopal et al. 2023), chimera states (Glaze et al. 2016), simulate sleep-wake cycle (Holmgren Hopkins et al. 2018; Qiu et al. 2021), and explosive synchronization driven by higher-order interactions (Farrera-Megchun et al. 2025b).

The goal of the paper is twofold: to show the firing patterns induced by the external current via nonlinear resonance, and to analyze the effects of resonance leading to coexisting attractors. We present evidence of a sequence of steps corresponding to frequency-locked states, which is shown in a Devil’s Staircase structure. These results are compared with the bifurcation diagram. Moreover, we observe the coexistence of three attractors, such as subthreshold oscillations, period-1 spiking, and chaotic spiking, or three periodic attractors, period-1 spiking, period-2 bursting, and subthreshold oscillation. We analyze these attractors using phase portraits, time series, bifurcation diagrams, and basins of attraction, the latter revealing a non-trivial structure with fractal properties that we quantify using the uncertainty exponent. To further verify the presence of chaotic dynamics, we compute the maximum Lyapunov exponent for representative attractors. Beyond their dynamical characteristics, we also investigate the energetic aspects of neuronal activity, showing how resonance-induced firing patterns and coexisting attractors differ in their levels of energy consumption. Studying these energetic properties provides additional insight into the functional relevance of resonance and multistability, particularly for the optimal transmission of signals (Li et al. 2024b; Yu et al. 2023).

The paper is organized as follows: Sect. 2 introduces the HB model, the model with an external current, and the range where the current will be studied. Section 3 presents the results obtained in three subsections: the effects on resonance (3.1), the effects on multistability (3.2), and the energetic analysis (3.3). Finally, Sect. 4 contains the discussions and conclusions.

## Model and methods

### The Huber–Braun model of thermally sensitive neurons

The Huber-Braun neuron model contains four variables, and is described as follows:

1 $$\begin{aligned} & C_{m}\frac{dV}{dt}=-I_{l}-I_{d}-I_{r}-I_{sd}-I_{sr}-I_{ext}, \end{aligned}$$

2 $$\begin{aligned} & \frac{da_{r}}{dt}=\frac{\phi (T)(a_{r_{\infty }}-a_{r})}{\tau _{r}}, \end{aligned}$$

3 $$\begin{aligned} & \frac{da_{sd}}{dt}=\frac{\phi (T)(a_{sd_{\infty }}-a_{sd})}{\tau _{sd}}, \end{aligned}$$

4 $$\begin{aligned} & \frac{da_{sr}}{dt}=\frac{\phi (T)(-\eta I_{sd}-\theta a_{sr})}{\tau _{sr}}, \end{aligned}$$

with

5 $$\begin{aligned} & I_{x}=\rho (T) g_{x} a_{x}(V-V_{x}), \ \ \ x=d,r,sd,sr, \end{aligned}$$

6 $$\begin{aligned} & a_{d}=a_{d_{\infty }}, \end{aligned}$$

7 $$\begin{aligned} & a_{x_{\infty }}=\frac{1}{1+exp(-s_{x}(V-V_{0_{x}}))}, \ \ \ x=r,sd,d, \end{aligned}$$

where $C_{m}$ is the membrane capacitance, *V* is the membrane potential, $I_{l}=g_{l}(V-V_{l})$ is the leakage current, and $I_{ext}$ is the external current. $I_{d}$ and $I_{r}$ are fast currents representing Na and K channels, respectively, which account for spike generation. $I_{sd}$ and $I_{sr}$ are the slow currents for subthreshold oscillations. $V_x$ is the equilibrium potential. The $a_{r}$, $a_{sd}$, and $a_{sr}$ are the activation channel variables for $I_{r}$, $I_{sd}$, and $I_{sr}$, respectively. The $a_{x_{\infty }}$ is the steady-state activation variable, with $s_{x}$ the activation slope and $V_{0_{x}}$ the half-activation potential. The constant $\eta$ takes into account the increase in calcium ions due to $I_{sr}$ and $\theta$ is the factor for the relaxation time constant. The constants $\tau _{r}$, $\tau _{sd}$, and $\tau _{sr}$ are characteristic times for the activation of $I_{r}$, $I_{sd}$, and $I_{sr}$, respectively. The model accounts for temperature effects by introducing scaling factors for both time constants and conductances, derived from the temperature coefficient $Q_{10}$.^1^ This approach follows the van’t Hoff principle describing the temperature dependence of reaction rates. Guided by experimental measurements in neurons and ion channels (Hille 1992; Hodgkin et al. 1952), the model adopts $Q_{10}=3.0$ for activation variables and $Q_{10}=1.3$ for maximum conductances. Accordingly, the temperature scaling factors are given by

8 $$\begin{aligned} \rho =1.3^{\frac{T-T_0}{10}}, \quad \phi =3.0^{\frac{T-T_0}{10}}, \end{aligned}$$

where *T* is the temperature, $T_0$ is the reference value, and $\rho$ and $\phi$ scale the maximum conductances and activation kinetics, respectively. This formulation ensures that the experimentally observed temperature sensitivity is consistently embedded in the model’s dynamics. All parameter values in the numerical simulation are shown in Table 1.

**Table 1:** Values of parameters used in the model. The system of unit is: capacitance in microfarads per square centimeter, conductances in millisiemens per square centimeter, potentials in millivolts, time constants in milliseconds, and temperature in degrees Celsius

Membrane capacitance $C_{m}$ = 1 $\mu F/cm^2$	
Conductances ($mS/cm^2$)	
$g_{l}$ = 0.1	$g_{d}$ = 1.5
$g_{r}$ = 2	$g_{sd}$ = 0.25
$g_{sr}$ = 0.4	
Equilibrium potentials (*mV*)	
$V_{sd}$ = $V_{d}$ = 50	$V_{sr}$ = $V_r$ = $-90$
$V_l$ = $-60$	
Activation time constants (*ms*)	
$\tau _{r}$ = 2	$\tau _{sd}$ = 10
$\tau _{sr}$ = 20	
Slopes of steady state activation ($mV^{-1}$)	
$s_{d}$ = $s_r = 0.25$	$s_{sd} = 0.09$
Half activation potentials (*mV*)	
$V_{0d} = V_{0r} = -25$	$V_{0sd} = -40$
Coupling and relaxation constants for $I_{sr}$	
$\eta = 0.012$	$\theta =0.17$
Reference temperature ($^{\circ }$C)	
$T_{0}=25$	$T=18$

### Neuron exposed to external perturbation

**Fig. 1:**
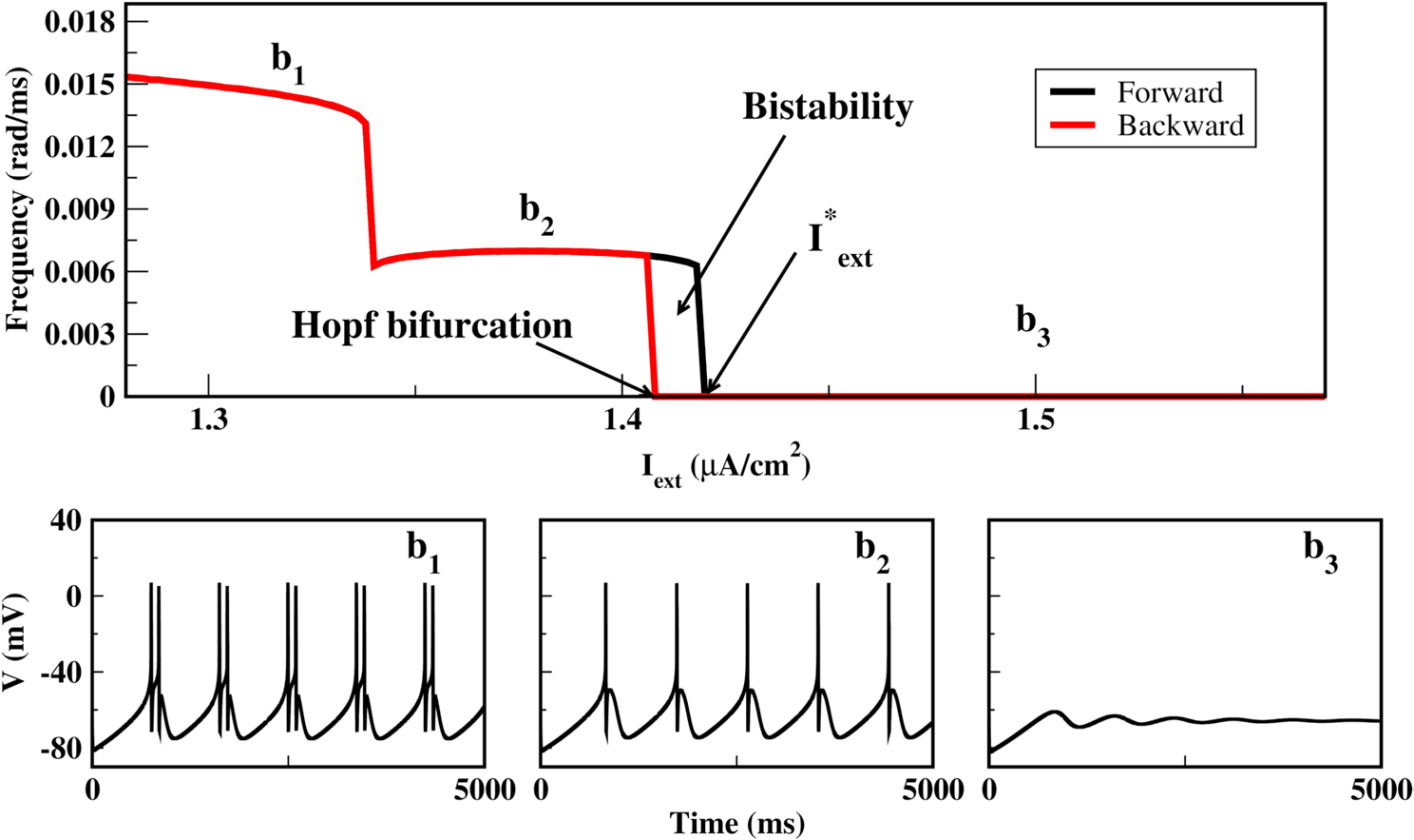
Firing frequency as a function of $I_{ext}$. Black (red) curve corresponds to the forward (backward) simulations, where $I_{ext}$ is increased (decreased) in steps of 0.002. We observe that for $I_{ext}>I_{ext}^{*}$ the neuron is in non-firing state (quiescent state) as is observed in $b_{3}$. There is a region of bistability and a Hopf bifurcation occurs in $I_{ext}\approx 1.4085$, and the firing patterns on the left side of the bifurcation point are shown in $b_{1}$ and $b_{2}$

We consider an HB neuron forced with an external current in a temperature of $18^{\circ }$C. The constant external current influences the firing dynamics, as shown in Fig. 1. The parameter $I_{ext}$ (in $\mu \text {A/cm}^2$) was varied using forward (increasing) and backward (decreasing) simulations in steps of 0.002, with the final state at each step serving as the initial condition for the next. In the backward simulation, the fixed point loses stability via a Hopf bifurcation at $I_{ext}\approx 1.4085$, giving rise to a stable limit cycle. In the forward simulation, the system remains on the limit cycle until it transitions to the quiescent state near $I_{ext}^{*}\approx 1.42$. Between these two values, both the fixed point and the limit cycle coexist. For $I_{ext}>I_{ext}^{*}$, the neuron always settles into the quiescent state.

To explore how the HB neuron model responds to the detection of a periodic subthreshold signal, we study the firing patterns of neurons stimulated by a sinusoidal perturbation:

9 $$\begin{aligned} I_{ext} (t)=I_{0}(1+B \text {sin}(\omega t)), \end{aligned}$$

where $$I_{ext}^{*} < I_{ext} (t)$$, meaning the $$I_{ext}(t)$$ oscillates in the non-spiking region. For a given $$I_{0}$$ value, the range of amplitude *B* is $$ 0< B < 1-I_{ext}^{*} /I_{0}$$, and $$\omega $$ is the forcing frequency with unit of rad/ms. In our numerical simulation, the fourth order Runge–Kutta method is used to solve the differential equations with time-step dt=0.01 ms together with the Julia package DifferentialEquations.jl (Rackauckas and Nie 2017).

## Numerical results

### Effects of external perturbation on resonance of a neuron.

**Fig. 2:**
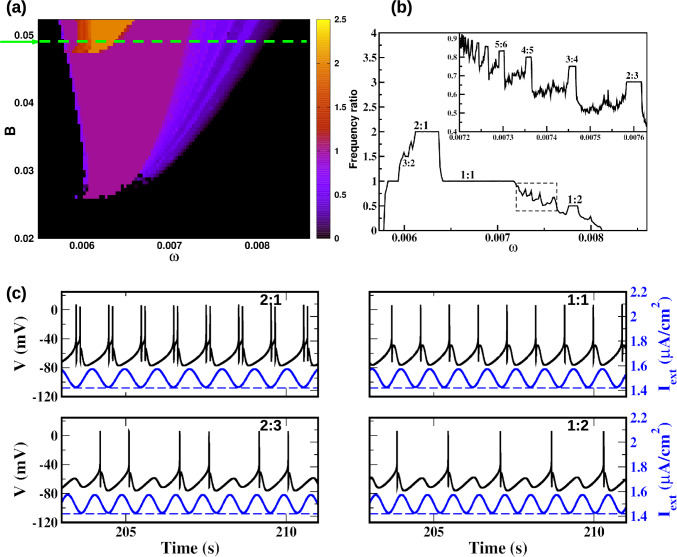
**a** Frequency ratio in the forcing amplitude *B* and external frequency $\omega$ parameter plane for $I_{0}=1.5$. **b** Frequency ratio as a function of $\omega$ for $B=0.049$. **c** Time series of the membrane potential for different situations of resonance 2:1, 1:1, 2:3, and 1:2. Also shown are the corresponding periodic modulations leading to their occurrence (in blue), and the dashed line is the critical value

We consider $I_{ext}=I_{0} (1+Bsin(\omega t)) > I_{ext}^{*}$, with $I_{0}=1.5$ $\mu \text {A}/\text {cm}^2$ and $0\le B \le 0.0523$. For $B=0$, the system exhibits steady-state dynamics, as shown in Fig. 1($b_{3}$). The frequency of damped oscillations near the stable focus is approximately 0.008263 rad/ms. We then explore different frequencies and amplitudes to scan the non-spiking region near the critical point in order to determine the parameter region where the spiking behavior for the forced systems is induced by resonance. The results are shown in Fig. 2.

In the color map of Fig. 2a, we plot the ratio of frequency between the firing frequency average of a neuron and the frequency of the forcing in the *B* and $\omega$ space. The colored region corresponds to parameter values where spiking activity is induced by the periodic forcing, whereas the black region indicates the non-firing state. The threshold separating these regions has a U-shaped profile, showing that resonance lowers the minimum forcing amplitude *B* necessary to generate spikes, with the strongest effect near the optimal forcing frequency. The optimal frequency is approximately 0.0061 rad/ms, which is close to the firing frequency (0.006267 rad/ms) near the critical current $I^{*}=1.42$. This value is also comparable to that associated with the dynamical behavior near the stable focus. Moreover, the perturbation induces different spiking dynamics in the resonance region. For example, for $B=0.049$ we observed different firing patterns corresponding to different frequency-locking states and it reveals a Devil’s-Staircase-like structure as shown in Fig. 2b. The time series for some phase-locked domains are shown in Fig. 2c.

**Fig. 3:**
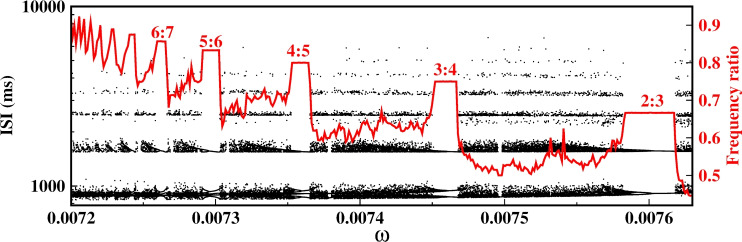
ISI Bifurcation diagram and frequency ratio as a function of $\omega$ for $B=0.049$

A specific region of the frequency ratio, marked by a dashed rectangle, and an enlarged view of this region is shown in Fig. 2b, and the Inter-Spike Interval bifurcation (ISI-bifurcation) is shown in Fig. 3. Here, a sequence of steps with relation $<f>=(n/(n+1))\omega$ is observed between $\omega =0.007174$ ($<f>=\omega$) and $\omega =0.007856$ ($<f>=\omega /2$) for *n* a positive integer. From the mathematical perspective, we know that if we have two rational fractions *p* : *q* and $p':q'$, the rational fraction that lies between them and has the smallest denominator is the rational fraction $(p+p'):(q+q')$, this is known as the Farey sum of the two rational numbers (Hardy and Wright 1979; Hilborn 2000). Therefore, we can understand the 2 : 3 locking as a rational fraction between lockings 1 : 1 and 1 : 2. The locking 3 : 4 is between 1 : 1 and 2 : 3, the 4 : 5 locking between 1 : 1 and 3 : 4, and so on. If we choose the rational 1 : 1 and $n:n+1$ for $n=1,2,3..$, we obtain the sequence observed in the Devil’s Staircase between the 1 : 1 and 1 : 2 frequency-lockings. We observe a bifurcation phenomenon characterized by alternating regions of resonance (discrete steps) and chaos. Each plateau corresponds to a periodic region. Furthermore, the numerator in the frequency ratio (*p* : *q*) indicates there are *p* branches that undergo period-doubling bifurcation to chaos, proceeding from right to left, e.g. in the 2 : 3 plateau, we observe two branches undergoing period-doubling bifurcation to chaos; in the 3 : 4 plateau, three branches; and so on.

The application of an external forcing modulates these behaviors, resulting in a structured chaotic pattern. Our results within the resonance region indicate that a dynamic of bursting with two spikes per burst, spiking of different periods and chaotic dynamics can be caused by a perturbation amplitude of the quiescent HB neuron.

### Coexisting attractors in resonance region

**Fig. 4:**
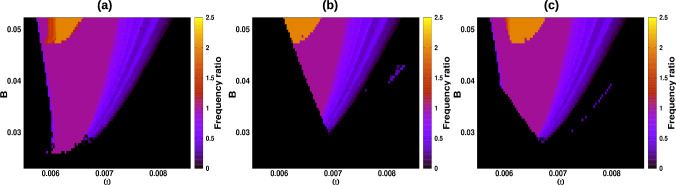
Frequency ratio in the forcing amplitude *B* and external frequency $\omega$ parameter plane for the following initial conditions: **a** (− 60, 0, 0, 0.5), **b** (− 60, 0, 0, 0.2), and **c** (− 20, 0, 0, 0.2)

The result of the previous subsection on the resonance region was obtained with a set of initial conditions, IC1=$(V_{0},a_{r_{0}},a_{sd_{0}},a_{sr_{0}})$=(-60, 0, 0, 0.5) Fig. 4a. However, we observed that the resonance region changed with other initial conditions. We calculated with two other condition sets, IC2= (-60, 0, 0, 0.2) Fig. 4b and IC3= (-20, 0, 0, 0.2) Fig. 4c. Different resonance states and firing patterns were observed, indicating that they depend on the initial conditions. In particular, we focused on the region $B=0.0475$ and $\omega \in [0.0058, 0.0066]$ where we observed the coexistence of three attractors.

**Fig. 5:**
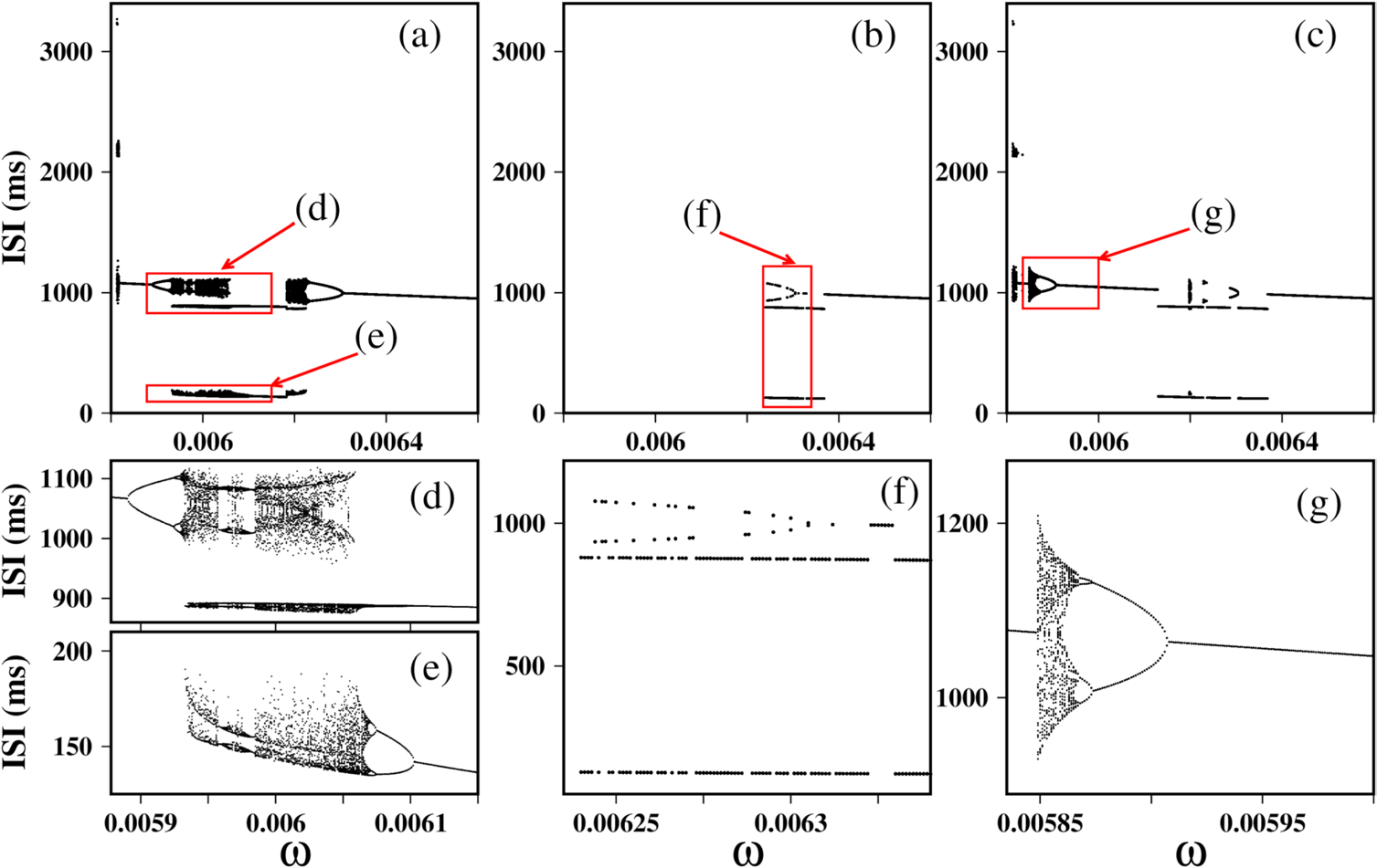
Bifurcation diagrams showing interspike intervals of the membrane potential as a function of $\omega$ for three initial condition sets: **a** IC1 = (− 60, 0, 0, 0.5), **b** IC2 = (− 60, 0, 0, 0.2), and **c** IC3 = (− 20, 0, 0, 0.2). Figures **d**–**g** are zoomed-in views of the highlighted regions in (**a**), (**b**), and (**c**)

We depict the ISI-bifurcations in Fig. 5 to further explore the firing patterns that can occur during the transition between electrical activities when we vary $\omega$ in the three sets of initial conditions. For the initial condition (− 60, 0, 0, 0.5) in Fig. 5a, the neuron exhibits different firing pattern transitions, for example, a transition from period-1 spiking to chaos via period-doubling bifurcation. This chaotic behavior is characterized by a mix of fast spiking, shown as low ISI values, and spiking with high ISI values. These fast spiking events are indicative of bursting behavior. Subsequently, the neuron undergoes periodic windows, such as period-2 bursting, period-2 spiking, and period-1 spiking. For the initial condition (− 60, 0, 0, 0.2) in Fig. 5b, we observed a non-firing state, followed by a range where the neuron changes between period-2 spiking and period-2 bursting as shown in Fig. 5f. Figure 5f presents a zoom-in of Fig. 5b. In this panel, the period-2 dynamics with ISIs around 1000 ms resemble those observed in Fig. 5a. However, Fig. 5f also reveals regimes not present in Fig. 5a, such as period-2 bursting characterized by long and short ISI values. The zoom-in highlights these transitions, showing more clearly how the dynamics alternate between period-2 spiking and period-2 bursting. For the initial condition (− 20, 0, 0, 0.2) in Fig. 5c, we observed a transition from period-1 spiking to chaotic spiking, followed by an inverse period-doubling bifurcation, and then a combination of spiking and bursting behavior.

**Fig. 6:**
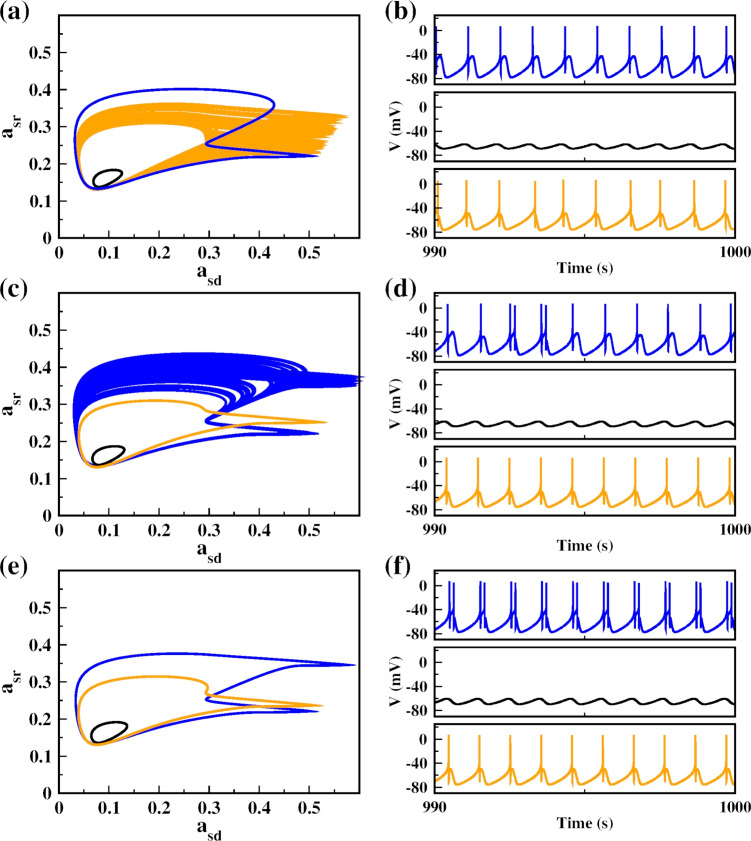
Coexisting attractors in the space $(a_{sd},a_{sr})$ on the left and the time series of the membrane potential on the right for 3 initial conditions: IC1 = (− 60, 0, 0, 0.5) in blue, IC2= (− 60, 0, 0, 0.2) in black, and IC3 = (− 20, 0, 0, 0.2) in orange. For **a** and **b**, $\omega =0.00585$; **c** and **d**, $\omega =0.006$; **e** and **f**, $\omega =0.00612$

In Fig. 6 we use frequency values of $\omega =\{0.00585, 0.006, 0.00612\}$ as examples to illustrate coexisting attractors in the $(a_{sd},a_{sr})$ plane and the time series of the membrane potential for the neuron under three initial conditions. For $\omega =0.00585$, we observe in Fig. 6a and b the coexistence of three attractors, period-1 spiking, subthreshold oscillations, and chaotic spiking. Figure 6c and d show three coexisting attractors: chaotic state with a mix of slow and fast spiking (bursting), subthreshold oscillations, and period-1 spiking. Finally, we also observe three periodic coexisting attractor as shown in Fig 6e and f.

The chaotic attractors identified in Fig. 6a and c are located in the dense regions of the bifurcation diagrams (Fig. 5), following a cascade of period-doubling bifurcations. To confirm their classification, we computed the maximum Lyapunov exponent ($\lambda$). For the attractors in Fig. 6a, $\lambda \approx 0.000475$ for the chaotic attractor, $\lambda \approx -0.001016$ for period-1 spiking, and $\lambda \approx -0.000698$ for subthreshold oscillations. For the attractors in Fig. 6c, $\lambda \approx 0.000392$ for the chaotic attractor, $\lambda \approx -0.000697$ for subthreshold oscillations, and $\lambda \approx -0.001699$ for period-1 spiking. The positive values confirm the chaotic nature of the corresponding regimes, clearly distinguishing them from quasi-periodic dynamics.

It is noteworthy that some resonance-induced attractors resemble those generated under constant current injection–for example, the period-1 and period-2 patterns in Fig. 6e–f are qualitatively similar to the spiking dynamics shown in Fig. 1b1, b2. However, nonlinear resonance also produces attractors absent in the constant-current regime, such as chaotic spiking–bursting states and subthreshold oscillations tied to the periodic perturbation. Thus, resonance both reproduces known behaviors and expands the repertoire of neuronal dynamics with qualitatively new attractors.

**Fig. 7:**
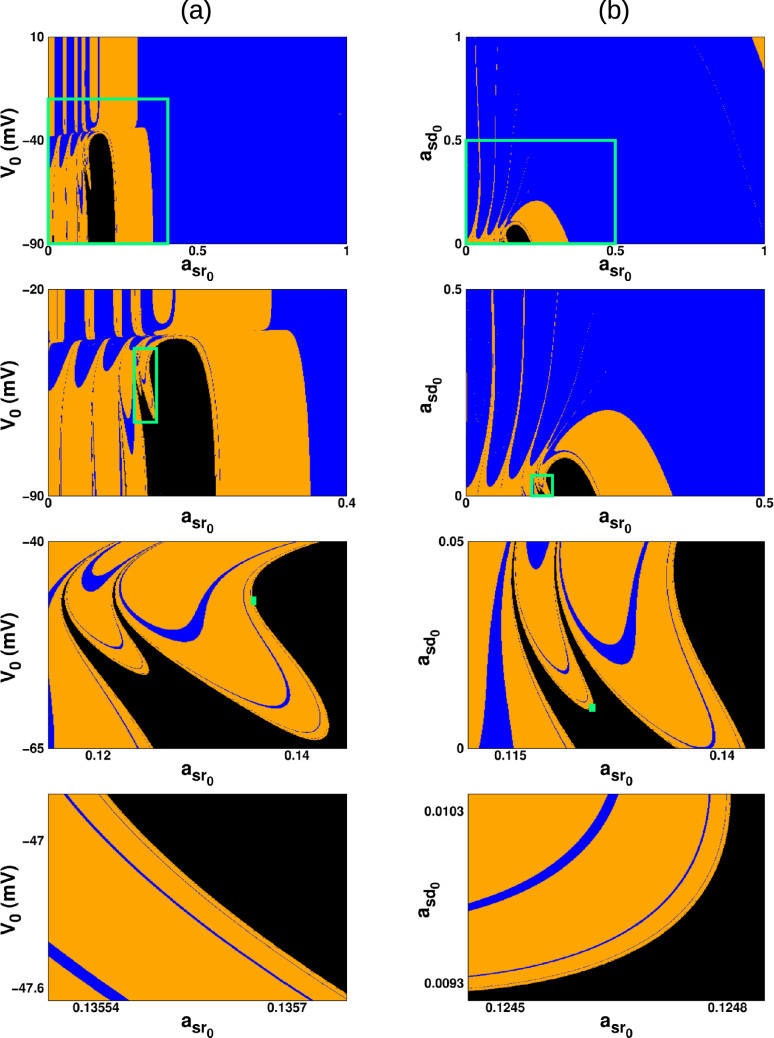
Basin of attraction for $\omega =0.006$ on the planes **a** $(a_{sr_{0}},V_{0})$ when all the other initial states are fixed at zero and **b** $(a_{sr_{0}},a_{sd_{0}})$ when $V_{0}=-60$ mV and $a_{r_{0}}=0$. The region in blue indicates chaotic behavior, orange indicates periodic behavior, and black indicates subthreshold oscillations. Zoomed-in views of the green rectangle are provided to more clearly reveal the structure of the basins of attraction

The basin of attraction resulting from a variation of the initial conditions of a neuron with external forcing of $B=0.0475$ and a frequency of 0.006 is shown in Fig. 7. We plotted the basin in the $(V_{0},a_{sr_{0}})$ plane (Fig. 7a) and the $(a_{sr_{0}},a_{sd_{0}})$ plane (Fig. 7b). The colors in this figure show the different starting points (initial conditions) that lead to the various firing activities seen in Fig. 6. Based on the basins of attraction, the area associated with chaotic firing appears greater than the combined areas for periodic firing activities and subthreshold oscillations. Additionally, we show some zoom-ins of this figure, which reveal hints of a complex basin boundary. This is, no matter how closely one examines a boundary point corresponding to subthreshold oscillations, all three basins appear in the detailed view, suggesting the emergence of a fractal-like structure.

**Fig. 8:**
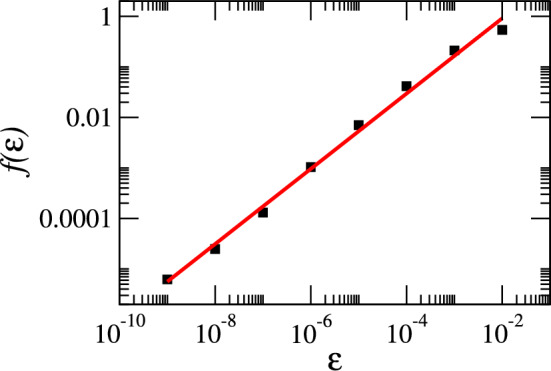
Log–log plot of the fraction of uncertain initial conditions $f(\epsilon )$ versus the perturbation size $\epsilon$ in the region $(a_{sr_{0}},a_{sd_{0}})\in [0.11,0.145]\times [0.0,0.05]$. The slope of the fitted line yields the uncertainty exponent $\alpha \approx 0.74464$

To quantitatively verify the fractal nature of the basin boundaries of the coexisting attractors, we estimated their fractal dimension using the uncertainty exponent method, which is widely applied in the literature (Bleher et al. 1988; McDonald et al. 1985). For this analysis, we focused on the region $(a_{sr_{0}},a_{sd_{0}}) \in [0.11,0.145]\times [0.0,0.05]$. For each initial condition in this region, we determined the corresponding attractor, and then compared it with the attractor obtained from the perturbed initial condition $(a_{sr_{0}}+\epsilon ,a_{sd_{0}})$, where $\epsilon$ is a small positive displacement. If the perturbed initial condition led to an attractor different from that of the unperturbed case, the point $(a_{sr_{0}},a_{sd_{0}})$ was identified as uncertain. The fraction of uncertain initial conditions, $f(\epsilon )$, was computed for each value of $\epsilon$ using all 160, 801 initial conditions. Repeating this procedure for eight values of $\epsilon$, we observed that $f(\epsilon )$ follows a power-law scaling, $f(\epsilon ) \propto \epsilon ^\alpha$, where $\alpha$ is the uncertainty exponent (if $\alpha < 1$, the basin boundary is fractal). Figure 8 shows the log–log plot of $f(\epsilon )$ versus $\epsilon$ together with the linear regression fit (red line). The slope yields $\alpha \approx 0.74464$. The fractal dimension of the basin boundary is then given by $d = D - \alpha$, where *D* is the dimension of the phase space (Bleher et al. 1988; McDonald et al. 1985). Since $D=2$, the estimated fractal dimension is $d \approx 1.25536$. This confirms that the basin boundaries of the three coexisting attractors are fractal.

### Energy considerations

Previous studies on the energy consumption of Hodgkin–Huxley neurons (Moujahid et al. 2011; Pal et al. 2021) have shown that the total energy in the circuit can be described as the sum of the energy stored in the membrane capacitor and that associated with the ionic batteries. Following this approach, we adapted the method for the HB model. The rate at which an ion channel supplies electrical energy to the circuit is given by the product of its electromotive force and the ionic current flowing through it. Accordingly, the total energy per unit time of the equivalent circuit in the HB neuron can be expressed as

10 $$\begin{aligned} \dot{H}=C_{m}V\dot{V}+I_{l}V_{l}+I_{d}V_{d}+I_{r}V_{r}+I_{sd}V_{sd}+I_{sr}V_{sr}, \end{aligned}$$

where $I_{l}=g_{l}(V-V_{l})$ is the leakage current, and $I_{d}, I_{r}, I_{sd}, I_{sr}$ are the ionic currents defined in Eq. 5.

Substituting the expression for the membrane voltage (Eq. 1) into Eq. 10 yields

11 $$ \begin{aligned} \dot{H} = & - VI_{{ext}} (t) - I_{l} (V - V_{l} ) \\ & \quad - I_{d} (V - V_{d} ) - I_{r} (V - V_{r} ) \\ & \quad - I_{{sd}} (V - V_{{sd}} ) - I_{{sr}} (V - V_{{sr}} ), \\ \end{aligned} $$

where the first term on the right-hand side represents the electrical power supplied by the external current, and the remaining terms correspond to the total energy consumption (metabolic energy) by the ion channels per unit time:

12 $$ \begin{aligned} E_{c} = & - I_{l} (V - V_{l} ) - I_{d} (V - V_{d} ) \\ & \quad - I_{r} (V - V_{r} ) - I_{{sd}} (V - V_{{sd}} ) \\ & \quad - I_{{sr}} (V - V_{{sr}} ). \\ \end{aligned} $$

The average energy consumption $\langle E_{c}\rangle$ of the neuron during its activity is then defined as

13 $$\begin{aligned} \langle E_{c}\rangle =\bigg | \frac{1}{T}\int _{t_{i}}^{t_{i}+T} E_{c}\,dt \bigg |. \end{aligned}$$

**Fig. 9:**
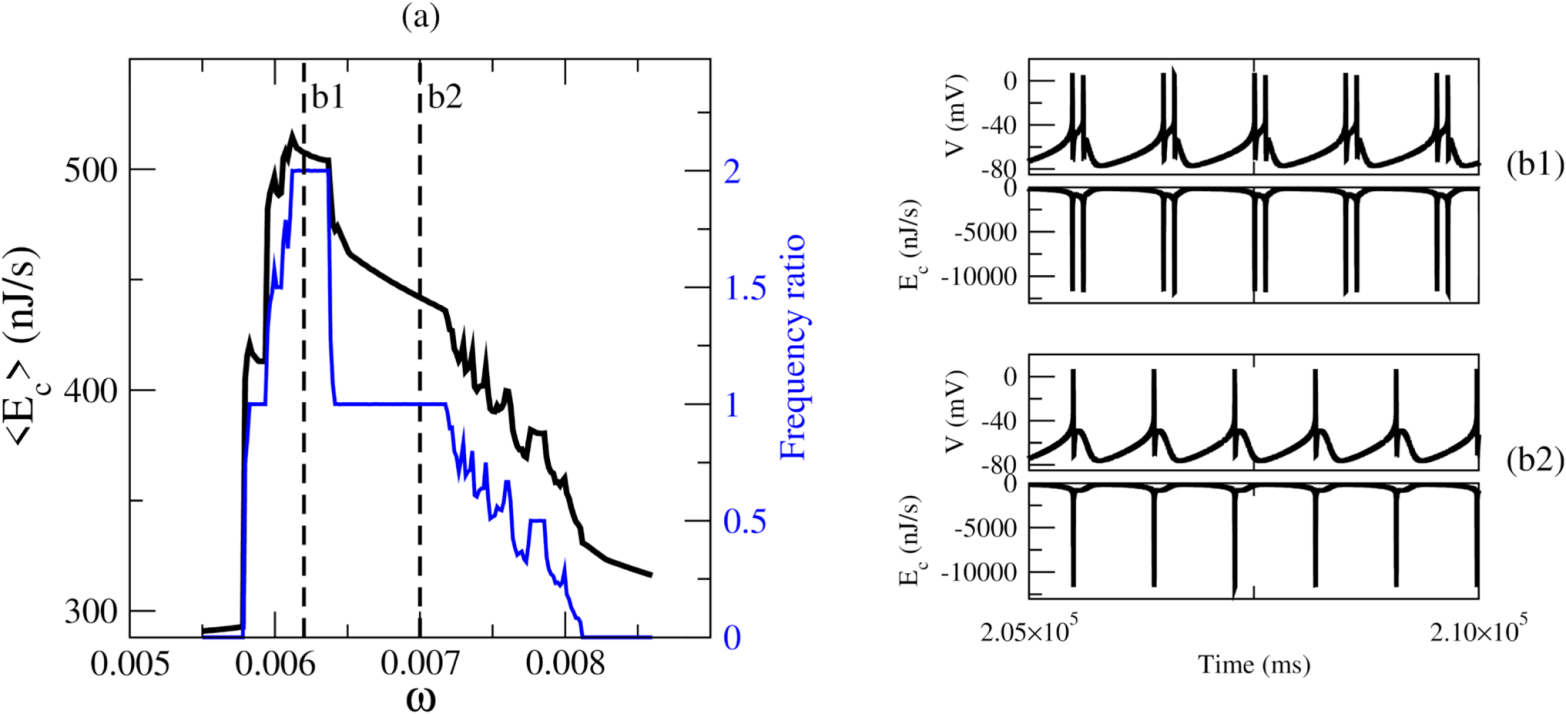
Energy consumption in the resonance region. **a** Average energy consumption as a function of external frequency and frequency ratio. **b1** and **b2** Time series of voltage and energy consumption for the 2 : 1 and 1 : 1 resonances, respectively

We computed $\langle E_{c}\rangle$ over a long simulation time $T=1\times 10^{6}$ ms with $t_{i}=5\times 10^{5}$ ms. Figure 9a shows the average energy consumption across the resonance region for $B=0.049$, together with the corresponding frequency ratio. We observe that resonance strongly affects the energetic requirements: the maximum occurs at the 2 : 1 resonance, followed by a decrease at the 1 : 1 resonance, after which the energy consumption varies non-monotonically as other resonance states and chaotic dynamics appear. Figure 9b1 and b2 illustrate the time series of voltage and $E_{c}$ for the 2 : 1 and 1 : 1 resonances, respectively. Moreover, when the neuron is quiescent at $B=0$, the energy consumption is approximately 285.76 nJ/s. By contrast, when the neuron is forced into a non-firing state with perturbation (e.g., $\omega =0.008135$), the energy increases to 330.22 nJ/s. This discrepancy arises because the HB model includes subthreshold currents, and under periodic forcing the resulting subthreshold oscillations in the voltage lead to additional energy dissipation.

**Fig. 10:**
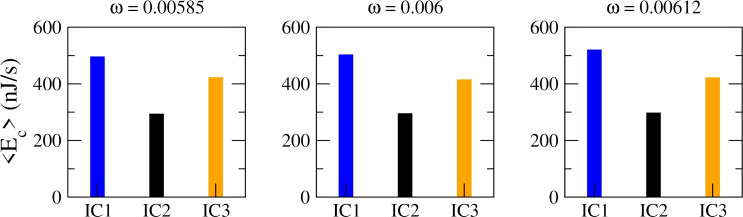
Energy consumption for the coexisting attractors observed in Fig. 6. **a** For $\omega =0.00585$, IC1: period-1 spiking, IC2: subthreshold oscillations, IC3: chaotic spiking. **b** For $\omega =0.006$, IC1: chaotic state (mixed spiking and bursting), IC2: subthreshold oscillations, IC3: period-1 spiking. **c** For $\omega =0.00612$, IC1: period-2 bursting, IC2: subthreshold oscillations, IC3: period-1 spiking

To compare the energetic requirements of different coexisting attractors (Fig. 6), we calculated their average energy consumption, with the results shown in Fig. 10. For $\omega =0.00585$, IC1 corresponds to a period-1 spiking attractor, IC2 to subthreshold oscillations, and IC3 to chaotic spiking. In this case, subthreshold oscillations consume the least energy, while chaotic spiking requires less energy than period-1 spiking. For $\omega =0.006$, IC1 is a chaotic state (mixed spiking and bursting), IC2 corresponds to subthreshold oscillations, and IC3 to period-1 spiking. Here, the chaotic attractor consumes more energy than the periodic attractor. Finally, for $\omega =0.00612$, IC1 is a period-2 bursting attractor, IC2 corresponds to subthreshold oscillations, and IC3 to period-1 spiking. In this scenario, period-2 bursting consumes more energy than period-1 spiking, which in turn consumes more than subthreshold oscillations.

Our results indicate that resonance and multistability likewise have energetic consequences. Specifically, changes in resonance states lead to corresponding changes in energy use. Among coexisting attractors, each exhibits a different level of consumption, and in some cases even chaotic firing consumes less energy than periodic activity. This suggests that multistability may enable neurons to balance energetic demands depending on their initial conditions and the influence of subthreshold perturbations.

## Discussion and conclusion

In this study, different firing patterns were induced by external stimulation through nonlinear resonance, by mean of which neurons are able to detect weak signal in the environment, including periodic and chaotic behavior. The incremental steps of this behavior result in fractal structures. One of them is the relation between the frequency of external current and the frequency of neuron, as it is seen in the Devil’s Staircase structure, and a particular segment follows the relation $n/(n+1)$ for *n* a positive integer. This relation has been observed in a silent Hodgkin–Huxley neuron exposed to sinusoidal electric field (Che et al. 2009). An interesting feature of this staircase is the interplay between periodic and chaotic dynamics. Each step is separated by a chaotic window. The importance of studying resonance in neurons lies on their ability to respond to low-amplitude forcing, which means it modifies their threshold.

A key finding of this study is the induction of multistability through nonlinear resonance, leading to the coexistence of different attractors. These attractors can include chaotic spiking, period-1 spiking, and subthreshold oscillation. The system can also exhibit the coexistence of period-2 bursting, period-1 spiking, and subthreshold oscillations. Because the system has no equilibrium point due to the time-dependent external perturbation, the attractors are hidden (Li and Sprott 2014; Bao et al. 2019). Recently, the coexistence of hidden attractors in nonlinear systems has attracted much attention as they produce a wide variety of complex dynamics (Dudkowski et al. 2016; Zhang et al. 2021; Chen et al. 2023). One effective method for exploring hidden attractors is through the analysis of their basins of attraction. Our observations, together with the calculation of the uncertainty exponent, indicate that the basin boundaries possess a fractal structure.

From a functional perspective, the coexistence of multiple attractors endows neurons with flexible response modes, allowing them to switch between different firing patterns under the same input conditions. Such multistability can act as a form of dynamic memory, where small perturbations drive transitions between stable states, thereby enriching the neuron’s capacity for information encoding (Breakspear 2017). Nonlinear resonance further enhances this capability by lowering the threshold for weak oscillatory inputs and introducing frequency selectivity, enabling neurons to preferentially respond to periodic signals at specific frequencies (Hutcheon and Yarom 2000; Pike et al. 2000; Gutfreund et al. 1995). In the specific case of temperature-sensitive neurons, resonance and multistability may act as complementary mechanisms for detecting weak periodic temperature fluctuations and flexibly encoding them into distinct firing patterns, as supported by the HB model and related studies (Braun et al. 1994; Finke et al. 2010b). Together, these dynamics expand the repertoire of neuronal responses and may underlie mechanisms for sensitive signal detection, reliable temporal coding, and information processing in sensory pathways, and may also contribute to synchronization in neuronal networks under fluctuating environmental conditions.

The phenomena described here are consistent with findings from various experiments. For example, Devil’s staircase-like sequences with step relations of $n/(n+1)$ have been reported in periodically stimulated squid giant axons (Takahashi et al. 1990). Subthreshold oscillations and resonance have been observed in hippocampal pyramidal neurons, entorhinal cortex and the amygdala (Pike et al. 2000; Hutcheon and Yarom 2000; Gutfreund et al. 1995; Pape et al. 1998). Multistability between distinct firing states has been documented in the R15 neuron of *Aplysia* (Canavier et al. 1994). These similarities suggest that the nonlinear resonance and multistability found in the HB model are not merely mathematical artifacts but may reflect mechanisms employed by real neurons.

In this study, the HB model was selected because it is a biologically inspired, conductance-based model that reproduces a wide range of firing patterns observed in neurons, including tonic spiking, bursting, chaotic dynamics, and subthreshold oscillations (Braun et al. 1998). The latter arise from the incorporation of slow currents (e.g., a simplified Ca-dependent K current) (Finke et al. 2010b), which are known to influence neuronal behavior in several brain regions, such as the entorhinal cortex and the amygdala (Gutfreund et al. 1995; Pape et al. 1998). This feature is not captured by the classical Hodgkin–Huxley (HH) model, making the HB model particularly suitable for exploring the effects of subthreshold perturbations. Specifically, the HH model reproduces spiking responses but does not generate subthreshold oscillations, as it lacks the slow currents that underlie such dynamics (Hodgkin et al. 1952). The HB model overcomes this limitation by incorporating these currents, which allow it to display subthreshold behavior and resonance-induced transitions between dynamical states (Huber and Braun 2006; Braun et al. 2003). Nevertheless, the model does not include all physiological details–for example, it lacks certain ionic currents (chloride, explicit calcium) and inactivation mechanisms–which may also shape neuronal responses. The novelty of the present work lies in demonstrating the emergence of complex resonance structures and the coexistence of distinct attractors induced by nonlinear resonance under subthreshold periodic stimulation.

Despite these insights, several key questions remain unanswered. The mechanisms underlying the emergence of hidden attractors in neural systems require further investigation, as does the interaction between distinct multistable states. Moreover, the role of network topology in shaping multistability remains an open problem. In this study, we selected $T=18^{\circ }$C because at this temperature the coexistence of three attractors was observed, after a preliminary exploration at different temperatures. While this suggests a potential influence of temperature on multistability, a more comprehensive investigation is needed to fully understand its effects on multistability and resonance in neurons. Here, we focused on the effects of subthreshold perturbations and the resulting dynamics rather than on the effects of temperatures. In addition, the HB model employed here does not incorporate explicit self-adaptive mechanisms; our analysis focused on multistability and nonlinear resonance under fixed parameters. Extending the model with adaptive dynamics (e.g., spike-frequency adaptation) would be an interesting direction for future work.

Recent reviews highlight the relevance of resonance and multistability for weak-signal detection in neurons and neural networks. For example, Calim et al. (2021) summarize how stochastic and vibrational resonance enhance signal propagation in complex neuronal topologies, while Large et al. (2023) review dynamical models of rhythm perception that emphasize phase- and frequency-locking as organizing principles across scales. Other recent surveys stress the role of energetic efficiency and controllability in biophysical neurons and synapses (Ma 2023). Building on these perspectives, several open problems remain: (i) how energetic constraints shape resonance and multistability at the single cell and network levels; (ii) how physiological parameters such as temperature reorganize coexisting attractors; and (iii) how network structure and feedback coupling enable signal amplification and synchronization (Liu et al. 2025). Addressing these issues will help connect dynamical models with functional roles in neural computation.

Beyond their theoretical significance, our results may have practical implications for neuroscience. Several observed patterns–such as resonance-induced spiking and Devil’s staircase-like structures–are consistent with experimental findings, whereas the coexistence of three attractors, though not yet reported biologically, provides predictive value by suggesting novel regimes under subthreshold forcing. Such resonance-induced multistability offers a potential mechanism for flexible information encoding, as neurons can switch between spiking, bursting, and subthreshold oscillations in response to weak inputs. More broadly, adopting a nonlinear dynamics perspective with models such as the HB neuron enables systematic parameter exploration directly linked to physiology, yielding predictions that can guide future experimental studies. An important possibility for future work is the experimental verification of these results. Electrophysiological recordings of temperature-sensitive neurons could test the predicted resonance-induced multistability, providing an opportunity to directly integrate computational and experimental findings.

## Data Availability

No datasets were generated or analysed during the current study.

## Footnotes

1 The $Q_{10}$ factor expresses the temperature sensitivity of a biological or chemical process, indicating how much its rate changes for every $10^{\circ }$C variation (Sterratt 2015).
